# Sensor technologies for quality control in engineered tissue manufacturing

**DOI:** 10.1088/1758-5090/ac94a1

**Published:** 2022-10-27

**Authors:** Mary Clare McCorry, Kenneth F Reardon, Marcie Black, Chrysanthi Williams, Greta Babakhanova, Jeffrey M Halpern, Sumona Sarkar, Nathan S Swami, Katherine A Mirica, Sarah Boermeester, Abbie Underhill

**Affiliations:** 1 Advanced Regenerative Manufacturing Institute, Manchester, NH 03101, United States of America; 2 Chemical and Biological Engineering and Biomedical Engineering, Colorado State University, Fort Collins, CO 80521, United States of America; 3 Advanced Silicon Group, Lowell, MA 01854, United States of America; 4 Access Biomedical Solutions, Trinity, Florida 34655, United States of America; 5 National Institute of Standards and Technology, Gaithersburg, MD 20899, United States of America; 6 Department of Chemical Engineering, University of New Hampshire, Durham, NH 03824, United States of America; 7 Materials Science and Engineering Program, University of New Hampshire, Durham, NH 03824, United States of America; 8 Electrical and Computer Engineering, University of Virginia, Charlottesville, VA 22904, United States of America; 9 Department of Chemistry, Dartmouth College, Hanover, NH 03755, United States of America; 10 Scientific Bioprocessing Inc., Pittsburgh, PA 15238, United States of America

**Keywords:** tissue engineering, regenerative medicine, process analytic technology (PAT), organoid, measurement, tissue engineered medical product, biosensor

## Abstract

The use of engineered cells, tissues, and organs has the opportunity to change the way injuries and diseases are treated. Commercialization of these groundbreaking technologies has been limited in part by the complex and costly nature of their manufacture. Process-related variability and even small changes in the manufacturing process of a living product will impact its quality. Without real-time integrated detection, the magnitude and mechanism of that impact are largely unknown. Real-time and non-destructive sensor technologies are key for in-process insight and ensuring a consistent product throughout commercial scale-up and/or scale-out. The application of a measurement technology into a manufacturing process requires cell and tissue developers to understand the best way to apply a sensor to their process, and for sensor manufacturers to understand the design requirements and end-user needs. Furthermore, sensors to monitor component cells’ health and phenotype need to be compatible with novel integrated and automated manufacturing equipment. This review summarizes commercially relevant sensor technologies that can detect meaningful quality attributes during the manufacturing of regenerative medicine products, the gaps within each technology, and sensor considerations for manufacturing.

## 1. Measurement and sensing for cells, tissue and organs

Engineered cells, tissues and organs offer enormous opportunity for the treatment of injury and disease. Over the last several decades, groundbreaking therapies for cell, tissue, and organ regeneration have shown success in small-scale research studies but a limited number of tissue engineered medical products (TEMPs) have translated into clinical practice [6]. Beyond the clinic, engineered tissues such as organoids, microphysiological systems (MPSs), and organ-on-a-chip systems offer promising solutions for disease modeling, drug screening, *in vitro* diagnostics, and cultured meat. The lack of translation is partly due to the high level of complexity of these products, high cost, and limited understanding of how the manufacturing process impacts product quality. Current manufacturing techniques utilize labor-intensive, mostly non-standard, and time-based process steps due to a lack of fit-for-purpose instrumentation and a vast number of undefined variables [7, 8]. These practices contribute to the high cost of production and batch-to-batch variation. While automation can be used as a tool to address operator introduced variability, controlling for the inherent biological variability of a living product requires specialized sensor technologies for in-process insight to ensure consistent product quality [7]. Even donor-to-donor variability could be monitored and controlled through a prudent sensing strategy. While cell and tissue product developers agree that the implementation of sensors is critical to progress the field, there are many limitations preventing the use of sensors in the manufacturing process. The Advanced Regenerative Manufacturing Institute conducted a community survey and found the most commonly cited reason for not measuring a target parameter in-process is a lack of a commercially available tool and lack of knowledge to implement. This article aims to provide a state-of-the-industry overview of sensor technologies for cell, tissue, and organ manufacturing, in hopes to (a) inform TEMP developers what manufacturing-ready sensor technologies are available and (b) outline sensor needs and design considerations for further development. This review is intended to outline the needs specific to the tissue engineering, organoid, and organ-on-a-chip community and build upon existing reviews in tangential fields such as biopharma, gene therapy, and cell therapy [9–17].

The first step to the implementation of sensors for tissue quality control is the definition of the QTPP [3, 4, 18, 19]. During ideation and discovery, powerful measurement tools are available and should be leveraged to conduct deep characterization of the tissue and associated process. This discovery process is used to define the QTPP which identifies the CQAs or the physical, chemical, and biological property that ensures the safety and efficacy of the product. With a solid understanding of the final product, a developer can leverage process development tools, such as design of experiments to understand the relationship between the process and the final product. The aim of this approach is to define the process design space and identify critical materials attributes (CMAs) and critical process parameters (CPPs) that are essential to achieving a safe and effective product. A control strategy can then be built around monitoring and controlling those set points in the process to ensure CQAs are met every time. This approach to product design and process development is called quality by design (QbD). By leveraging a QbD approach, developers can integrate sensor technologies into a manufacturing workflow as process analytic technologies (PATs), increasing the understanding of process variables and allowing for better control and flexibility within the production [18, 20–23]. Sensor technologies play an essential role in identifying and controlling process variability, saving resources and time for developers, and making a more predictable product with supporting evidence that has an increased chance of meeting regulatory scrutiny.

**Figure 1.**
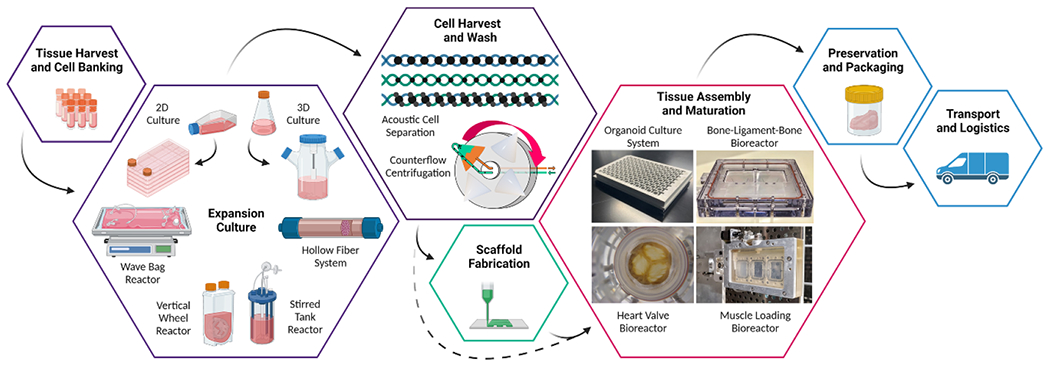
Steps of the cell and tissue manufacturing process. Many unique bioreactor form factors exist to expand cells (e.g. hollow fiber systems, stir tanks, wave bags, vertical wheels) and grow organs and tissues (e.g. bone-ligament bone (CGEM^™^ by STEL Technologies), heart valves (Aptus Bioreactors), muscle (TEMR by George Christ at Univ. of Virginia, bioreactor by DEKA Integrated Solutions Corp), or organoids (System by Tommy Angelini)) (graphic created with BioRender.com).

In the biopharmaceutical space, a QbD and risk-based approach that is empowered by the integration of in-process sensors and measurement has been heavily encouraged. In 2004, the US Food and Drug Administration (FDA) put forth guidance for the industry, encouraging the use of PAT and sensors to ensure effective and efficient manufacturing processes based on a mechanistic understanding of how the process factors affect product performance [19]. The pharmaceutical production space now has a robust array of commercial options for the detection of in-process characteristics (e.g. temperature, oxygen, pH, and suspended cell biomass concentration) and final product attributes [9, 10]. The recent advent of cell therapy manufacturing for cell and gene therapy products has underpinned the need for PAT that can detect cellular identity traditionally destructively assessed by measuring endogenous factors and gene expression [13]. The tissue and organ space share this need for detection of cellular attributes, however monitoring the liquid media phase may not provide insights into what is happening throughout the layers of cultured tissue. Furthermore, biopharmaceutical cell cultures are often large-volume homogeneous mixtures of suspended cells, and appropriate sensors developed to monitor conditions have limited application for cell, tissue, and organ therapies since, for example, cell expansion typically requires an adherent cell culture surface. A dynamic culture environment requires sensors that can interface with the cultivation across multiple, often heterogeneous form factors (figure 1) with local phenotype differences that could have important functional consequences.

Measurement and monitoring are needed throughout the manufacturing process for cells and tissues (figure 1), beginning with the harvest of raw materials such as the isolation and purification of stem cells from a patient source [24, 25]. The cells are then expanded and/or differentiated in tissue culture to reach target cell numbers and phenotype for tissue generation or incorporation into a scaffold. Factors such as donor health, age, and sex contribute to different growth rates. The cell population is then harvested from the expansion culture and concentrated for seeding into a scaffold or tissue maturation bioreactor. However, during the previous expansion step, a less desirable subpopulation may have dominated the culture and the culture no longer colonizes the chosen matrix efficiently. In many tissue manufacturing processes, cells are incorporated with a structural scaffold support material during scaffold fabrication (e.g. 3D printing, injection molding, electrospinning). After combining the components, the tissue is matured in a bioreactor that carefully controls and guides the development of the tissue. Sensors can be integrated during any of these stages as a means of in-process quality control, process monitoring, process gating or decision making, or real-time, automatic control.

**Figure 2.**
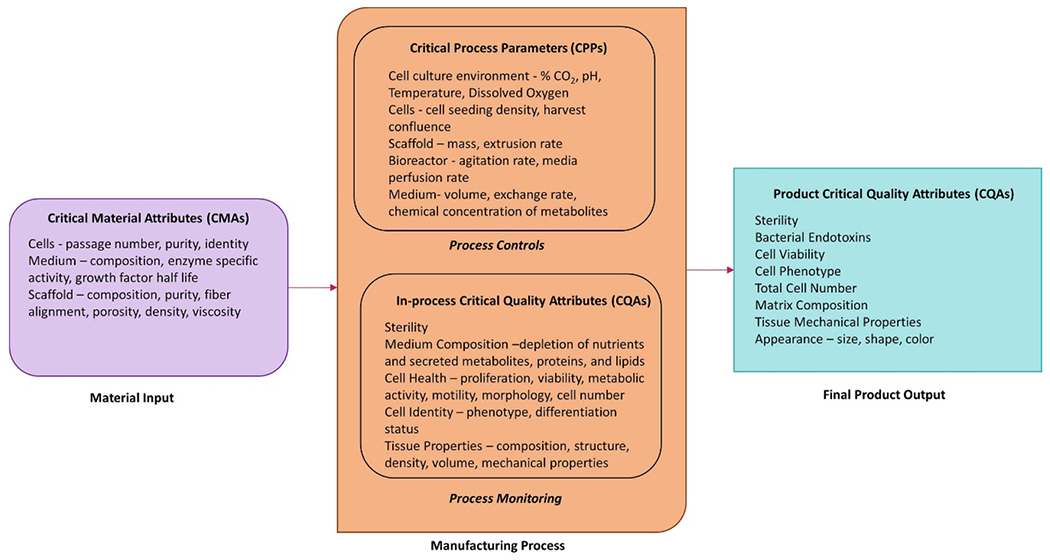
Example CMAs, CPPs, and CQAs that need to be measured in a manufacturing process.

**Figure 3.**
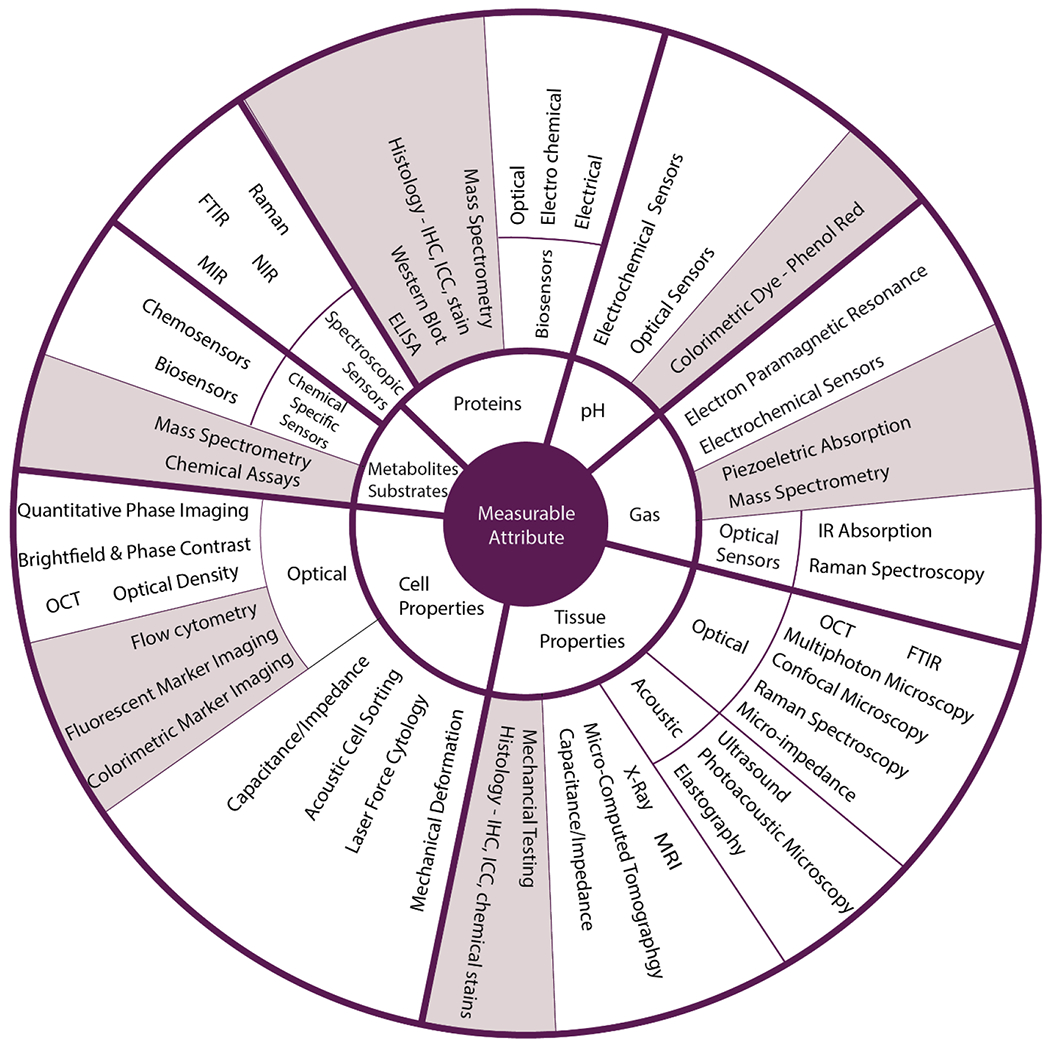
Measurement approaches to assess a given attribute. Highlighted areas identify measurement approaches that are not easily integrated into a manufacturing process because they are considered an off-line measurement or prohibitively destructive to the product. Non-highlighted areas have a product either in development or commercially available that is will meet the criteria of being at-, on-, or in-line sensor. This chart is meant as an overview; there may be other approaches or measureable attributes not included. Abbreviations: mid-infrared spectroscopy (MIR), near-infrared spectroscopy (NIR), Fourier transform infrared spectroscopy (FTIR), optical coherence tomography (OCT), magnetic resonance imaging (MRI), immunohistochemistry (IHC), immunocytochemistry (ICC).

During the manufacturing process, developers are interested in the in-process quality attributes that correlate to the final CQAs [26]. Integration of in-line sensors to detect in-process CQAs enables real-time release testing of the final product, ensures product quality throughout the process, and streamlines data generation for continued process and product understanding. Beyond the basic cell culture environment (pH, temperature, O_2_, CO_2_), quality attributes of interest to measure are indicators of cell health and identity, functional tissue properties, sterility, and the presence of nutrients and secreted metabolites, proteins, and lipids in tissues and culture medium (figure 2). Each of these attributes can be measured by an array of measurement approaches (table 1). For example, proteomic analysis can provide insight into the extracellular matrix proteins excreted by the cells indicating the material composition of the scaffold as well as cell phenotype. This review will describe available sensors for assessing (a) tissue properties, (b) cell properties, (c) proteins, (d) substrates and metabolites, (e) gases and volatile organic compounds (VOCs), and (f) pH (figure 3).

Sensor development and implementation require streamlined communication and collaborative work between traditionally distinct fields. The development of a process-integrated sensor requires: (a) the identification of a target analyte by a cell or tissue developer; (b) the development of a method to detect and quantify the analyte by the sensor and measurement developer; (c) the production of a manufacturable format by a manufacturing expert; and (d) interfacing of the newly developed sensor and measurement technology with a software and data acquisition platform by an automation or integration expert. The application of measurement technology into a manufacturing process requires the cell and tissue developers to understand how to appropriately apply a sensor into their process and for sensor developers to understand the design requirements and end-user needs of the cell and tissue manufacturing process.

## 2. Sensors and measurement considerations for manufacturing

Existing commercial solutions fail to meet several design requirements such as compatibility with manufacturing equipment, cost, scalability, accuracy, response time, or detection range [27]. Traditional measurement assays for evaluating the properties of cell and tissue products require manually obtaining the sample (i.e. culture media, cellular material, or tissue material), conducting the measurement process which is usually destructive and lengthy, analyzing the readout, and reporting the result. In this workflow, the product is altered by the post-process test method and therefore does not reflect the real-time status of the product. These approaches place a high burden on employee time, pose a sterility risk to the product, involve costly destruction of the product, and/or are not timely relative to its shelf-life. Measurement techniques (e.g. histology, blots, enzyme-linked immunosorbent assays (ELISAs), polymerase chain reaction (PCR)) that are manual, destructive and offline will not be discussed in this review. While these measurement tools are appropriate for discovery and small-scale research and development, they are challenging to implement in a scaled manufacturing process that requires an automated process workflow. The measurement techniques used to detect in-process attributes need to be non-disruptive to the process, cost-effective at a commercial scale, and provide data on a timescale appropriate to the system dynamics.

Sensors and measurement techniques implemented as part of a manufacturing workflow should minimally impact the system. Biological materials are highly sensitive to their environment; therefore, a measurement technique should minimize how much the measurement process interacts with the product or alters the cultivation environment (destructiveness). The interaction includes downstream effects of the technique on sample behavior and the volume of sample analyzed or extracted from the system. Furthermore, contamination from bacteria, fungi, endotoxin or mycoplasma poses a significant safety risk in patients and is highly disruptive to the cultivation of the product; therefore, a measurement technique should minimize contamination risk exposure (invasiveness). In the context of this review, destructiveness and invasiveness of a measurement technique are considered on a spectrum. For example, a tissue bioreactor could utilize force sensors to detect tissue mechanical properties in response to a controlled applied strain. The load would not destroy the tissue like a uniaxial pull to failure mechanical test, however, it would likely mechanically condition the tissue and trigger cells to respond to the load experienced from the test. Similarly, a Raman probe sterilized and immersed in a closed system for the duration of culture would be considered less invasive than frequent system interaction to draw samples from the otherwise closed process to be assessed on a benchtop metabolic analyzer. The long-term clinical effects should be carefully considered for any technique that permanently labels, marks, or tags the final product to be implanted in the patient.

To achieve fully automated in-process measurements, the sensor or measurement technique should be capable of detecting the target at a rate that reflects real-time system dynamics. A real-time measurement occurs on-demand or continuously with a response time faster than the time it takes for the dynamics of the system to change. This response time must be inclusive of the time taken to prepare and analyze the sample. Digital integration of these types of sensors at a minimum allows for process monitoring, while a more advanced implementation would be continuous process control as part of a feedback loop or PAT.

**Figure 4.**
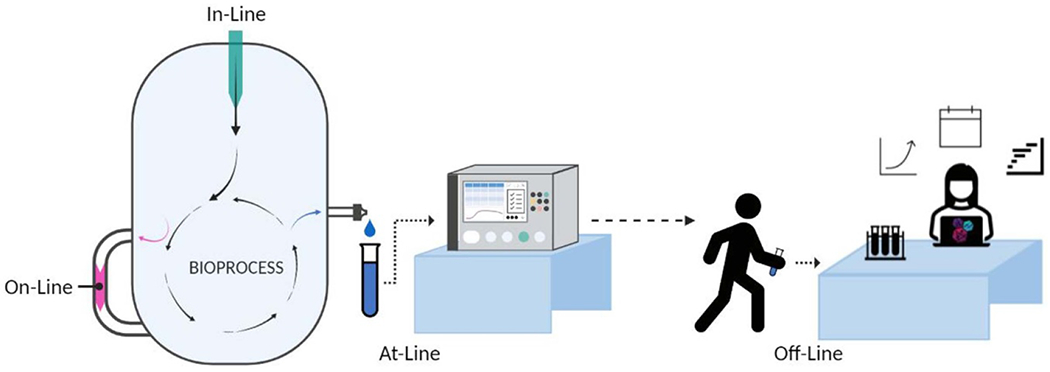
Diagram illustrating in-line, on-line, at-line, and off-line measurements in a TEMP bioprocess (graphic created with BioRender.com).

Physical and digital integration of sensors into the manufacturing process is essential to achieve real-time monitoring and control. Sensors are classified as in-line, on-line, at-line, and off-line (figure 4). An inline measurement does not remove the sample from the process stream, detecting the target directly within the system [5]. An on-line measurement diverts the sample from the manufacturing process to an analytical instrument and the sample may or may not be returned to the process stream [5]. An at-line measurement removes or isolates the sample to be rapidly analyzed in close proximity to the process stream [5]. This contrasts with an off-line measurement in which the sample is removed, isolated from, and analyzed in an area remote from the manufacturing process [5]. The location of the sensor or where the sample is extracted within the system will also impact how the reading is interpreted (i.e. local vs global, specific vs bulk).

The high degree of complexity of a cell or tissue product means that several sensors will likely be required to interface with the system. Hence, with limited space, when possible, sensor developers should consider combining several sensors into a multimodal or multiplexed sensor system. In addition, many different bioreactors are used and so sensors will need to be flexible to accommodate different container materials (figure 1).

When implementing a sensor technology, the product developer should consider the quality of the measurement technique to ensure that the measurement method meets its intended use and a certain degree of reproducibility [28, 29]. This means the measurement technique should be assessed for precision, accuracy, sensitivity, repeatability, and quantitation range [4, 5, 30, 31]. Additionally, the specificity or ability to assess unequivocally the analyte in the presence of components that may be expected to be present should be considered [31]. The technique should be robust enough to remain unaffected by small variations in parameters under normal usage [31]. The user should understand how each step of the measurement process (i.e. preparing, processing, and analyzing) can introduce variability and where to apply process controls. Performance specifications should define acceptable ranges and deviations, and reference materials to provide control values for targets should be utilized when available [28]. These metrics will ensure that the measurement reading is a true reflection of the value and is consistent within a production run as well as across multiple sites, different production runs and different pieces of the same analytical equipment.

For any measurement, the user needs to clearly define any assumptions being made for that measurement and how the data will be used in decision making [32]. The frequency and volume of the sample determine whether a statistically meaningful result can be used to inform decision-making. When conducting a measurement, the user assumes the technique is going to inform the quality attribute. For example, the use of a bioimpedance measurement assumes a particular capacitance level for a viable cell with an intact membrane, but this threshold can vary based on cell death mechanism. To calculate the viable cell number from viable biomass, there is an assumption on the average mass per cell. The analytical algorithm for measurement analysis can also make assumptions, with or without the knowledge of the user. While often these automated algorithms reduce user variation during analysis, the user should be aware of how the measurement is analyzed and any errors that may propagate as a result.

The ability to implement any sensing technology is often driven by the cost to implement and maintain the technology. The calculation of cost is multifactorial. With an automated and integrated sensor, the cost of manual labor hours should be minimized to maximize the skills of the operator. The cost depends on the number of uses or lifetime of the sensor, as well as whether the sensor components are durable or consumable. Developers should be wary of over-engineering an automated solution when a low-cost disposable sensor can be equally effective without pricing out users. By using a QbD approach, developers can simplify the quality control strategy through specific low-cost sensors rather than over-whelming data processing with complex information. It is a common pitfall with the advent of computer-aided technologies to collect more data than is reasonable or necessary.

Similarly, the question of scale should be carefully considered. Costly instrumentation and imaging platforms are limited to monitoring a single culture system. For example, automated cell confluence imagers would be considered on-line, real-time, non-destructive, and non-invasive. However, these systems are limited to monitoring only one culture flask at a time (e.g. Lux 2 by CytoSMART, Paula by Leica, CM20 by Olympus). Automated systems that deliver the culture system to the analytical instrument so that multiple systems can be monitored on a schedule is one way around this limitation. There are commercial solutions available for automated media draws (e.g. BioProfile Flex2 On-line Autosampler by Nova Biomedical, Automated Sampling System by Flow-namics, BioPAT Trace by Sartorius) as well as culture hotels that deliver culture dishes to imaging instrumentation (e.g. BioSpa by BioTek, BioStation CT by Nikon). These systems tend to be cost-prohibitive, limited to small culture volumes, and require the cultivation system to be specifically designed to accommodate that workflow. Alternatively, some systems will network an array of probes across several cultivation systems that report to a single instrument for processing and analytics.

Sensor integration into a manufacturing process means the measurement workflow, to the extent possible, is automated. Ideally, an automated sensor would be less susceptible to measurement variability as a result of the workflow; however, users should consider if any controls need to be put in place to ensure a quality measurement (e.g. sampling, sample prep, data collection, data processing and analysis) [33, 34]. With the increasing application of algorithmic analysis, artificial intelligence (AI) and machine learning (ML), the user needs to be aware of assumptions made in an algorithm and how an error could potentially propagate. Furthermore, while data are easy to obtain, it is not cheap to store high volumes of data and the quality of the data is the essential ingredient to meaningful computer-aided analysis. This underscores the importance of high-quality measurement at the outset. Depending on the extent of model building, a significant amount of investment of resources may be required to implement automated PATs, investment in this type of technology should be considered as early as possible to maximize return on investment.

When reviewing the sensors described in the following sections, the user needs to think carefully about the measurements fitness for purpose. Fit-for-purpose evaluation includes the (a) intended use of the measurement (b) considerations for the properties of the sample (c) consideration regarding the measurement method and (d) any assumptions made regarding the measurement method or sample [29, 34].

## 3. Sensors to detect meaningful quality attributes

**Figure 5.**
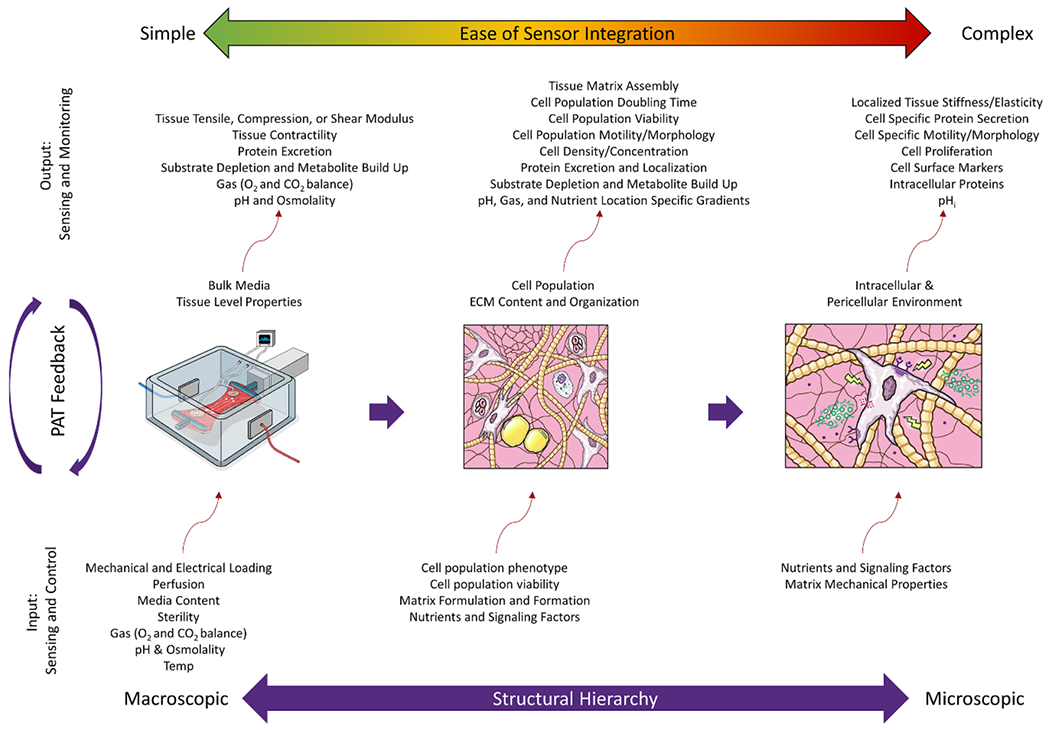
Sensing of an attribute is a data snapshot within a complex dynamic multi-scale system. Sensing is required as both an input and output of the system and can be representative of an attribute across different structural scales. The difficulty of conducting a measurement increases as the environment that is being sensed gets smaller and the measurement more specialized. Any attribute measurement must be contextualized within the full spectrum of the tissue culture system.

In the world of regulated medical products, TEMPs are unique in that their mechanism of action is likely a combination of biochemical and mechanical functions to achieve efficacy. The functional properties of a tissue are a culmination of interactions between cells, scaffold materials, and chemical signaling factors. Integrated sensors within the tissue manufacturing process provide insight into the complex set of interactions (figure 5). At a basic level, growth of any tissue will require sensing the metabolic process through the exchange of nutrients and waste and cellular respiration through the conversion of O_2_ to CO_2_. These baseline metrics of cell health are relatively easy to detect in the bulk culture media using existing technologies. Given the highly specialized cell types and diversity of tissue properties, more specialized sensors are required to detect targets reflective of cell phenotype and tissue properties. These sensors are less available on the commercial market and more complex to integrate into a manufacturing process. Furthermore, the properties that need to be assessed range from macroscale tissue properties to microscale cellular properties and chemical signaling through proteins, metabolites, substrates, and other small molecules. A systems-level approach will better define the mechanistic parameters involved in the biological outcome and develop an effective sensing approach [35]. The following sections overview manufacturing relevant sensors available for the detection of quality attributes pertaining to tissue properties, cell properties, proteins, metabolites, gases, and pH.

### 3.1. Tissue properties

#### 3.1.1. Overview and significance

The composition, structure, and function of a tissue is frequently the critical attribute to evaluate during the development or final evaluation of a TEMP. The composition may influence cell phenotype including matrix production and remodeling *in vitro*. Additionally, the tissue composition profoundly influences the way a patient’s body will respond once implanted. When developing a TEMP, the extracellular matrix, cell type, cell density, and chemical signaling factors that compose the product are spatially and temporally dynamic. Tissues are most often heterogeneous in composition and elucidating that variance may be essential to the tissue’s performance in its intended use, whether that be to treat, diagnose or model a disease. Furthermore, the structure and function are tightly linked, as the mechanical integrity of a tissue is essential to its physical performance. Both macroscopic and microscopic structure not only directly impacts the physical behavior of the tissue but drives cellular behavior [36]. Developers should be aware of the spatial resolution (sub-cellular, cellular, micro, macro) and depth in which they are obtaining information. Tissue properties will vary by location and microscale properties are not always representative of macroscale behaviors. For these reasons, non-destructive methods to assess tissue properties throughout and at the conclusion of culture are needed for TEMP production.

#### 3.1.2. Sensor technologies

Current commercial methods to measure tissue properties assess the physical spatial compositions in 3D, focusing specifically on the composition and structure of the TEMP. These methods, such as histology [37], immunohistochemistry [38, 39], scanning electron spectroscopy [40], and transmission electron microscopy [41] are terminally destructive where the tissue is cut and chemically processed, involve lengthy manual processing and therefore are not real-time or in-line. Other functional assessments, including mechanical and electrical testing, are generally end-point assays that require destructive testing or if they are non-destructive the tissue is heavily manipulated, altering the final product and posing a significant sterility risk to the product while in process. There is a need for methods beyond the destructive mechanical testing, histology, and immunohistochemistry that better integrate with manufacturing processes and provide more real-time feedback on CQAs.

**Figure 6.**
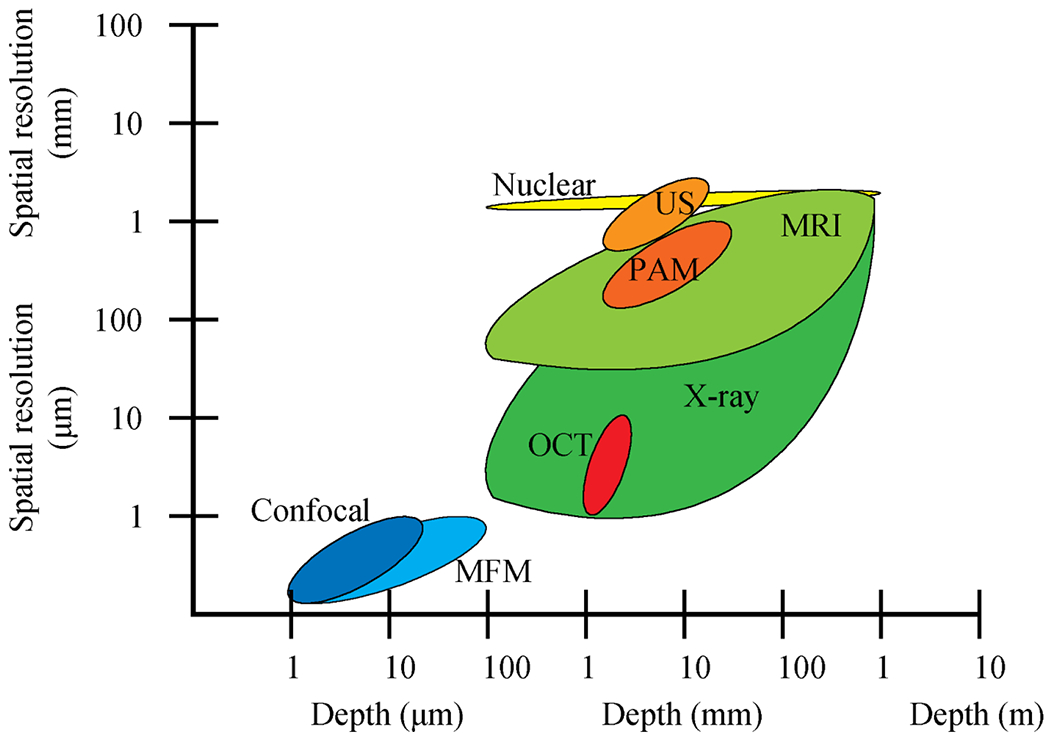
Approximate ranges of spatial resolution and imaging depth achievable by imaging modalities for tissues. US = ultrasound, PAM = photoacoustic microscopy, MRI = magnetic resonance imaging, MFM = multiphoton fluorescence microscopy, OCT = optical coherence tomography. Reprinted from [46], Copyright (2013), with permission from Elsevier.

The methods that align with better manufacturability are, for the most part, still in research and development for application to TEMPs. When looking at tissue structure and composition, different types of imaging approaches show the most progress for tissue property characterization. Imaging depth, contrast, field of view, and spatial resolution are measurement considerations that dictate which type of technology best fits a TEMP and the overall manufacturing process. Optics-based non-destructive evaluation of healthy and pathological tissue has been shown to produce corroborative and congruent results when compared to traditional histology [36]. This includes multi-photon microscopy [42, 43], which uses the adsorption of photons by a fluorophore to allow real-time observation of single cells and molecules in tissues, confocal microscopy [44], which stacks high-resolution images using a pinhole to focus illumination and detection optics to create 3D reconstructions of tissues, and optical coherence tomography (OCT) [45], which measures echo time delay versus the magnitude of backscattered light [46, 47]. These rapid and label-free techniques have varying imaging depths and resolutions, and characterize a tissue’s matrix composition and structure, as well as cell distribution and morphology (figure 6) [46, 48]. Another type of imaging includes elastography, where different imaging modalities combine to show soft tissue properties. These methods include, as optical coherence elastography [49, 50], an OCT-based elastography detecting depth-resolved sample deformation; holographic elastography [51], which images acoustic wave propagation to calculate material properties; and ultrasound elastography [52], which monitors the response of a sample to acoustic energy [46]. Ultrasound imaging is a rapid, label-free, and quantitative vibrational spectroscopy method that can elucidate the new formation of tissue matrix, structure and function of vasculature, and viscoelastic properties when coupled with optical imaging and elastographic tools [46]. When combined with optical image contrast, ultrasound creates photoacoustic microscopy (PAM) [53], which is scanning-focused ultrasonic transducer based and has better imaging depth than some optical techniques [46]. Electrochemical impedance spectroscopy is an emerging technique that uses the electrical conductivity of a sample to determine scaffold and tissue properties [54]. The ability to simultaneously apply multiple frequencies provides contrast for porosity and conductivity properties [54–57]. Two-dimensional laser displacement can also be used to measure scaffold porosity, but this technique also has applications in measuring matrix composition in 3D bioprinting [58]. X-ray imaging approaches, including MicroCT (*μ*CT) and x-ray Phase Contrast, which measure variations in absorption, refraction, and scatter properties of x-ray, are another technology that shows promise for investigating tissue properties. X-ray phase contrast can show tissue structure, biomaterial structure, and foreign body response, while *μ*CT can show the internal structure of scaffolds within a TEMP [46]. Magnetic resonance imaging (MRI) [59], which uses magnetic resonance signals with magnitude and phase to depict anatomical features, and then Raman spectroscopy [60–62] and Fourier transform infrared (FTIR) spectroscopy [63], which use vibrational spectroscopy to probe molecular changes, are techniques also in development for use in characterizing tissue structure and composition. FTIR applies light at several wavelengths simultaneously onto a sample, with the reflected or transmitted light then measured in the form of an interferogram. With Raman, the shift in the color of the light incident onto a sample is measured. While many of these approaches are commercially available as at-line or off-line instruments, the main challenge with deploying these approaches in-line is sterile access to the culture environment and scaled deployment. These approaches are highly sensitive to the focal plane, liquid medium content and level as well as the material (e.g. bioreactor wall) to image through. The tissue bioreactor would likely need to be designed to accommodate the specific needs of a detection approach. The scalability of these approaches is limited due to the high cost of core instrumentation. Tissue samples would need to be delivered to the instrument to be used cost-effectively, limiting ease of use and frequency of detection.

While functional conclusions can be drawn from various imaging techniques, direct functional characterization of a TEMP may be required to assess the desired functional attribute. Mechanical loading and sensing technologies can show real-time changes in mechanical properties for a TEMP. One example of a relevant technology in development is a microphysiological strain gauge that is created via multi-material 3D printing [64]. This device uses piezo-resistive and biocompatible soft materials that detect contractile stresses in cardiac tissue. Another example is an impedance-based measurement that uses platinum polydimethylsiloxane pillar-shaped electrode to measure extracellular field potential, also originating at contractile stresses from *in vitro* cardiac tissue [65]. Embedded force sensors [66] and electrochemical measurements are both techniques that have also been utilized in characterizing TEMP mechanical properties. These approaches are in the research and development stage, but have the potential to be more easily integrated than optical approaches. Since these sensors would contact the tissue, the sensor material cytotoxicity and how the sensor contacts the tissue should be carefully considered. Disparate mechanical properties in contact with tissues could influence tissue behavior and development over time as well as contact could limit nutrient diffusion. Another consideration is the detachment of the sensors from the tissue at the conclusion of the culture.

There are some commercially available products on the market that help characterize mechanical properties. Increasingly, companies are developing mechanical characterization tools specific to tissues that can be used as part of a culture system (e.g. TA Instruments, CellScale Biomaterial Testing and Biomomentum, Mantarray^™^ by CuriBio). The non-destructive in-line mechanical characterization will require further development and eventual standardization before it could be deployed as part of a manufacturing process.

#### 3.1.3. Outlook

While all of the aforementioned technologies show potential in characterizing tissue properties, there are many challenges to be addressed before application within manufacturing. Data analytics and interpretation from sensor responses prove difficult when many of the technologies extract spectrum and time-dependent data. Since many of the measurement technologies rely on multiparametric forms of sensing, it increases the complexity for data analysis for continuous measurement.

As more TEMPs enter the commercial arena, non-destructive technologies that integrate into the manufacturing process will be essential. Currently there are commercially available instruments but they are used off-line or designed for clinical use. The bulky size and high cost associated with much of the non-destructive instrumentation is a major barrier. Additionally, many of the approaches rely on a highly-skilled worker to conduct the analysis. Multimodal devices will need to be miniaturized and downsized in the form factor of sensors [36]. These technologies could run into challenges with shifting to in-line monitoring if the sensing platform cannot interface with the tissue bioreactor. There is also an outstanding need for standards development in the space to enable better translation, increase safety and reliability, improve in-process efficiency, and decrease the overall costs of TEMP biomanufacturing [36, 67]. Overall, the tissue property sensor space has many opportunities. Addressing challenges with translating the technology to readable and useful data within a manufacturing process would greatly improve TEMP manufacturing and make more usable and commercially available technologies.

### 3.2. Cell properties

#### 3.2.1. Overview and significance

Cells are often considered the central workhorse in the development of a tissue construct and their biological activity is tightly linked to the identity, potency, purity, and safety of a TEMP. Properties of cells, including mechanical, optical, electrical, and vibrational properties can serve as indirect measures of functional and biological attributes. Some biophysical properties include their size, shape, deformability and electrical physiology, including that of their microenvironment and of each of their subcellular regions (membrane, cytoplasm, organelles, nucleus, etc) [68]. Importantly, the biophysical properties of cells and their microenvironment can provide valuable label-free information on cell attributes [69] making them a particularly promising class of measurements in closed cell manufacturing processes and unit operations. For example, cell morphology and cell volume have been associated with mesenchymal stromal cell differentiation [70, 71]. Additionally, cell health also influences biophysical properties [72], necrosis often causes swelling, while apoptosis causes shrinkages and secretion of smaller subcellular bodies, which can be monitored to predict transformations of adhered cultures [73]. The biophysical properties of a cell are routinely leveraged to conduct baseline measurements of cell number, concentration, and viability in a cell, tissue, and organ production process. These metrics are typically CPPs for achieving desired cell seeding density, harvest confluence, and cell purity as well as often critical to determining the final purity and potency of the product or normalizing other sensor outputs.

#### 3.2.2. Sensor technologies

Cell count, cell viability and overall cell health are common quality attributes that need to be assessed for any cells that will serve as starting materials for TEMPs. There are a number of at-line and off-line approaches that utilize the biophysical properties as well as the biochemical properties of cells to inform on these quality attributes. At-line approaches typically use dye exclusion membrane permeability assays with manual microscopy, flow cytometry, or dedicated cell count and viability analyzers (e.g. Chemometec NucleoCounter, Nexcelom Cellometer, Beckman Coulter Vi-cell, GeminiBio Moxi V) [74]. These assays utilize dyes that are destructive and not easily amenable to on-line or in-line sensing. Recent advances in at-line cell count and viability analyzers utilize label-free approaches based on cell impedance to evaluate cell viability (e.g. Cellix Inish Analyser). Other measures of cell viability, that are generally more informative of overall cell health (e.g. apoptosis assays, metabolic assays and cell proliferation assays), are typically conducted off-line as they can require more time, sample handling, and sample preparation [34]. For example, immunocytochemistry to assess intracellular and cellular surface markers falls under this umbrella, requiring labeling and may destroy and contaminate the cell sample preventing subsequent use. These methods typically require manual processing of the cells before analysis (i.e. removing adherent cells from their culture surface) and utilize dyes that require permanently labeling the sample and recording a fluorescent, colorimetric, or luminescent signal. An important exception is the resazurin assay, which provides a metabolic marker of cell viability and can be incubated directly with a culture or TEMP to report cell metabolic function in living cultures.

On-line and in-line sensors are now in-development and commercially available that will assess cell count, viability, and cell health. These sensors tend to fall into four broad categories (a) optical, (b) electrical, (c) mechanical, and (d) vibrational.

The most common label-free biophysical measurement modality is optical imaging. Label-free quantitative phase imaging, which quantifies the phase shift of light through a cell, has provided biophysical measurements that can be associated with cell viability [75, 76]. Instruments such as the Ovizio cell analyzer can connect to a bioprocess, capture cell images, and then utilize image analysis and ML techniques to extract information correlated to cell health or other cell functions. Instruments such as the Cytosmart and Incucyte can sit directly in a cell culture incubator and capture continuous images of cells and organoids as they grow to evaluate morphological features of cells and cell confluence. Others have shown that optical tracking of cell motility and morphology along with automated intelligent analytics yield comparable results to end-point staining such as immunohistochemistry (Nikon BioStudio T). However, these approaches tend to be limited to cell culture in 2D (i.e. plates, dishes, flasks) or small cell aggregates/organoids and relatively low throughput, limited to monitoring one imaging area at a time. For 3D culture, optical density approaches use backscatter measurements to monitor the biomass non-invasively through the glass wall of the bioreactor (Aquila Biolabs BioR). Optical density measurements work well on homogeneous suspended particles but, struggle to deliver reliable readings on cells cultured in aggregates or on the surface of microcarriers.

The biophysical behavior of cells in the presence of an electrical field can be used to measure basic attributes such as cell shape, and size, as well as more complex metrics such as cell phenotype [77–79]. Viable biomass can be calculated non-destructively and in real-time using capacitance and impedance measurements (e.g. Aber Futura, BioPAT^®^ Viamass). Cells with an intact membrane screen the applied electrical field at low frequencies but pass it to the interior at high frequencies, with the capacitance of cells in the medium providing information on the number density of viable cells. Unlike optical approaches, which can be obscured by cultivation solids such as microcarriers or extracellular matrices, the electric field can penetrate these materials at high frequencies (>MHz). Application of this technology in a flow-cell type format can achieve single cell resolution; however, probes for bioreactors require a minimum cell density and total volume to occupy the electrical field to detect a difference from the background. To make conclusions about the cell number, biomass to cell count conversation assumes that all cells are the same size and density. This approach becomes less reliable if the cell population contains heterogeneous cell types or cells that may variably cluster.

Cellular response to mechanical stresses, such as deformation due to fluid flow, or passage through microfluidic channels, has also been used to classify cells for cell health, differentiation status, or viral load [80–82]. For example, commercial instruments exist that can assess membrane stability and cell functionality of blood cells through the deformation of cells under shear stress and osmotic conditions (e.g. Lorrca). The size and deformation of cells can be used to design microfluidic chips to detect, focus, mix, count, lyse, and analyze individual cells on an integrated platform [83] (e.g. Cytorecovery Cyto R1). Additionally, microchip devices are well suited for parallelization and present a label-free approach that can also sort and measure individual cells, without diluting the enriched fractions for off-chip analysis. Cell sorting on microchips provides numerous advantages over conventional methods by reducing the size of necessary equipment, eliminating potentially biohazardous aerosols, and simplifying the complex staining protocols commonly associated with cell sorting. However, application is often limited by their ability to scale-up the isolation of sufficient cell numbers required for transplantation. Laser Force Cytology combines microfluidics and optics together to identify, characterize, and sort cells (e.g. LumaCyte Radiance). Although these techniques are well suited for characterizing the cellular starting materials for TEMPs, the primary disadvantage to applying mechanical approaches to TEMPs themselves is that the cells must typically be removed from their culture surface and analyzed in a single cell suspension.

Similar to mechanical approaches, the vibrational properties of cells can be used to conduct cell characterization and sorting. Traditional flow cytometry has been modified or augmented to realize new functionalities. Flow cytometry has also been combined with imaging techniques to assess morphology alongside surface marker analysis (Amnis^®^ ImageStream^®^) [84]. Acoustic cell processing has emerged as a new tool that uses acoustofluidics principles [85] to clarify, perfuse, concentrate, wash and select cells all in a label-free and centrifuge-free process (FloDesignSonics ekko). The size, density, and compressibility of the cells under acoustic forces capture cells in a standing wave while fluid flows through the device. Acoustic cell sorting can be used for higher volume analysis in a cell processing work stream; however, optimization is required to achieve the sensitivity to specific cells types in particular media [86–88].

#### 3.2.3. Outlook

Many commercially available approaches are either destructively conducted at-line or require cells to be non-adherent in a single cell suspension. Cells for tissue and organ applications will likely be embedded in a scaffold material, on the surface of a material such as a microcarrier, or in dense aggregates or cell layers. OCT is emerging as biomedical imaging technique that can perform cellular-resolution imaging *in situ* and in real-time. Research studies have shown that OCT can be used to assess cell viability [89] and cellular dynamics such as migration and proliferation within scaffolds [90]. There are commercial products in development that deploy OCT in-line to detect cell concentration, aggregation level, and cell viability (e.g. ChromoLogic). However, commercial approaches will need to be developed for characterizing adherent cells in or on a support material. Similarly, impedance-based approaches are being explored for cell characterization within scaffolds or on surface materials. For example, dielectric impedance spectroscopy combined with supervised ML can be used for non-destructive assessments of cell type and maturity within tissues [91, 92]. Impedance-based image reconstruction [93] of cell architectures within 3D cultures has been undertaken to study structures of spheroids [94] and neurons [95], while spectral methods have been used to quantify cellular processes at single-cell sensitivity, including activation [96], migration [97] and differentiation [98]. These models and analyses are highly sensitive to the material and cell type and need to address challenges with heterogeneous material and cell types before implementation into a manufacturing process.

Many of the available approaches can sufficiently detect viability and health however fall short of cell phenotype. Raman-based flow cytometry has been explored as a rapid, label-free, and non-invasive analytical technique to assess cell membrane properties and assess cell cycle dynamics [99–101]. Advancements in the application of AI and ML into image-based approaches is a promising approach. Another emerging method for cell phenotype analysis is impedance-based flow cytometry [102] which provides multi-parametric information on each sub-cellular region based on the frequency of the applied field (0.1–100 MHz). For instance, the identity of cells can be related to membrane conformation and folds, determined from capacitance measurement at low frequency (1–5 MHz), while information on the cytoplasm and nucleus can be obtained at high frequency to identify stem cell subpopulations [102]. Recently this method was utilized to directly analyze adhered cells on microcarriers to assess their numbers and viability [73].

Cellular biophysical measurements typically yield data of low dimensionality (i.e. signals along with fewer functional attributes), which has limited its wider application. Hence, recent advances are focused on multiparametric approaches for high throughput biophysical analysis of single cells, coupled with ML models for automated phenotypic classification of its information rich content and for in-line phenotypic recognition to trigger downstream steps (e.g. sorting or drug treatment) [103–105]. Key gaps include the computational power required for recognition (especially the case for image data and less so for electrical and mechanical data), the lack of rigorous cell standards for data normalization to compare across biological samples, and the availability of relevant control samples with defined phenotypes [106, 107] for training ML models towards classification and neural network algorithms towards in-line recognition [108]. Additionally, label-free biophysical methods are not typically direct measures of a cell’s function or health and are rather a summarized reflection of more complex cellular processes. This can make the interpretation of biophysical properties challenging further emphasizing the importance of appropriate training data.

Finally, although biophysical measurements are label-free, the forces and perturbations (e.g. light exposure, mechanical forces, electrical stimulus) used to probe biophysical properties could have consequences on the cells and tissue under development. Close monitoring of the samples will be needed in the development of characterization methods using biophysical methods with experimental designs to test for the unintended consequences of seemingly non-destructive methods.

### 3.3. Proteins

#### 3.3.1. Overview and significance

The health of cells, cell phenotype, composition of the organ and tissue, and the growth phase of a culture can be monitored by measuring the concentration of specific proteins [109, 110]. Cell, organ, or tissue maturity and functional attributes can be measured by using different proteins as biomarkers [111, 112]. Sensors can be incorporated in-line to directly measure the quantity or concentration of a protein biomarker in the medium [15, 22, 113]. Protein measurements can also indicate when contamination occurs or when cells are dying or multiplying [114, 115]. Measurement of the concentration of a protein will give direct feedback to the operator and the system for improved quality control. For example, protein measurements can be used to determine when it is time to switch to another phase of growth, such as determining if cells are mature enough to use fatty acids instead of glucose [116] or assessing stages of cell and tissue maturity [117]. Protein measurements are important for both process optimization and quality control; the growth process and feedstocks can be modified at just the right time to optimize the quality, throughput, and the yield of the growth process.

#### 3.3.2. Sensor technologies

While there are many different types of sensors that can measure protein concentration, two different types are described here: 1) spectroscopic, namely the technique to look at a spectroscopy profile to identify a chemical fingerprint for the protein, and 2) protein-specific sensors, namely biosensors with a recognition element for selectivity with different transduction modalities [118]. These two types of protein biosensor encompass most of the protein sensing important for cultivation of cells, tissues, and organs.

Spectroscopic sensors, such as those based on infrared (IR) and Raman spectroscopy, can be designed for non-invasive, in-line or on-line, continuous monitoring of media for optically active species [23, 119, 120]. IR measurements include FTIR, near IR (NIR) and mid IR which are IR techniques that measure different wavelengths in the IR. One advantage of this approach is that they can be trained to detect one or several target proteins, depending on the complexity of the environment. Many spectroscopic sensor probes can be used directly in a bioreactor chamber (in-line) or a small, on-line perfusion chamber via transmission without coming in direct contact with the growth medium. The intensity of the light is typically at a low enough energy to not disturb, disrupt, or destroy the protein sample. Industrial examples of in-line and on-line process sensors include Merck-MilliporeSigma Pro-Cellics Raman Analyzer, Kaiser Optical RXn2, Repligen CTech^™^ FlowVPE^®^ System, Nirrin Technologies RTA-2300, MarqMetrix Raman AIO analyzer and FlowCell^™^, and Tornado HyperFlux Pro Plus.

While spectroscopic sensors are capable of continuously and simultaneously measuring multiple analytes, significant challenges remain before meeting the needs of TEMP manufacturing, including selectivity. Automated spectroscopic measurements rely on algorithms to interpret the data effectively, yet proteins can have similar optical properties and overlapping signals [121, 122]. Big data and ML techniques aid in extracting specific protein information, yet this process is more difficult if an unknown protein is introduced or if many different types of proteins are present in the sample [123–128]. The need to provide training data to the algorithm for each measurement setting is also a challenge. While advancements have been achieved, selectivity and specificity remains a primary concern of spectroscopic sensing. The primary disadvantages of spectroscopic-based sensors are their relatively high cost; the requirement for data sets to train the algorithm that extracts concentration information (several replicate cultivations with both on-line monitoring and off-line sample analysis); and the complex and opaque data analysis that may be a concern for regulators if transparency is required [129]. These factors make spectroscopic sensors better suited for production cultivations (in which the goal is to perform the optimal process conditions repeatedly) than for smaller-scale, process development operations. These non-specific sensors are generally successful for lifetime, stability, and in-line implementation but would be more widely used if calibration were simpler and cost were lower. Adding a second, more specific laser or adding an optical transduction tool can improve selectivity, but these options remain expensive [130].

Protein-specific biosensors use a biological recognition element (e.g. antibody, aptamer, or single-strand DNA) to gain detection specificity via a targeted binding event [118]. The biological recognition element is typically attached to a surface, and the binding event results in a transducible signal. Different transduction systems exist, including optical (e.g. fluorescence, surface plasmon resonance (SPR), surface-enhanced Raman spectroscopy), electrochemical (e.g. voltametric, amperometric), and electrical (e.g. impedance, resistive, field effect transistors). However, at the time of writing this article, all current protein biosensors are designed for offline or at-line use [131]. One common assay format employs a recognition element attached to beads and a tag used in a flow cytometry format to measure several specific protein concentrations (e.g. Luminex 100/200). Other commonly used protein biosensors are florescence tags with fluorescence properties that change in the presence of the protein (e.g. Triage by Quidel). Electrochemical systems have cheaper transduction systems than their optical counterparts [132] and several electrochemical transduction biosensor examples are available, including Simoa HD-1 Analyzer by Quanterix, Portable Cardiac Reader and D-Dimer Test Cartridge by Zepto, and Photoelectric ELISA by Advanced Silicon Group.

The strength of sensors using biological recognition elements is the specificity of the measurements. Users should bear in mind this specificity and assess if they are detecting protein fragments or larger fully functional proteins. Current technologies using electrochemical measurements require sampling of the chamber and are considered minimally invasive [131]. These biosensors require the analyte specific surface to be replaced, which limits in-line or on-line use of biosensors. Strategies to rejuvenate the surface (i.e. to decouple the protein analyte from the surface) have been explored, but typically use highly troublesome chemicals that would usually disrupt the culture growth [133, 134]. Use of protein biosensors in the at-line format requires a small volume sample be extracted to conduct the measurement. For most systems, this does not negatively impact the cell, tissue or system dynamics. Some companies have invested in automation of at-line protein biosensors, but this level of automation (sampling and sensor use) can be expensive and cost restrictive, and increases volume requirements for use in small bioreactors.

#### 3.3.3. Outlook

Typically, when proteins are targeted, there is a tradeoff between spectroscopic-based assays, which are currently multiple-use and in-line or on-line, vs protein-specific assays, which have cheaper equipment (upfront) cost with higher specificity. While both spectroscopic and biosensor techniques show promise for use with TEMPs, the user needs to know which proteins to target. Resolution, response time, precision, reusability, and selectivity are extremely important protein sensor considerations for cell, tissue, and organ processes. For most applications, current technologies are capable of sensing proteins at a sufficient lower limit of detection. Current protein biosensors are capable of detecting their target analyte to provide a null/present determination in a single-use modality; however, a null/present output lacks the quantitative capability to detect fluctuations in protein levels. The current technology for protein-specific assays requiring off-line analysis have a limited capability of measuring real-time changes in protein levels within culture media or tissue, therefore, limiting the power at-/in-/on-line protein measurements could have in improving quality of products in the development of TEMPs remains to be proved.

Looking forward, both spectroscopic measurements and biosensors show promise for detecting specific proteins in cell, tissue, and organ cultures. For spectroscopic measurements, there is work in combining different measurement techniques, such as multi-modal measurements, which will have enhanced specificity over single modal measurements [115, 135]. In addition, there is work in improving the algorithms for analyzing spectroscopic measurements to obtain more specificity. Other efforts have looked at how to apply complex instrumentation, such as mass spectrometry, for in-line or on-line real-time analysis [136]. For proteomic applications, ML and big data techniques are being used to support measurement of 20–100 proteins simultaneously via mass spectrometry [137–139]. As in-line or on-line proteomic approaches become more cost-effective and selective, ML will make a larger impact in bioprocessing. In addition, groups are exploring alternative biological recognition elements that can easily be regenerated without the need for invasive chemicals [ 140]. This work includes using nanostructures to have improved sensitivity, easing the cost-performance trade-off, using stimuli-responsive surfaces, and making multiplexed testing systems that test for many protein concentrations in one test [141].

Most of the methods described in this section address protein sensing in the medium rather than in the tissue itself. The current technology state of protein-specific biosensors to assess cell surface markers, intracellular proteins, or tissue composition, cell or tissue needs to be extracted from the system and destructively assessed. Destructive assessment maybe a viable approach for cell and tissue specific targets if replicate samples can easily be obtained such as 3D cell culture on microcarriers, cell aggregates, or organoids. Researchers are exploring the use of genetically encoded reporters that activate a colorimetric or fluorescent indicator when a transcriptional pathway is triggered [142, 143]. A few spectroscopic measurements, such as Raman, can measure proteins directly in tissue [144, 145]. Second harmonic generation using multiphoton microscopy, Raman spectroscopy, and FTIR are in development for localized detection of specific proteins within a tissue [60–62, 144–146] These offer the advantage of being non-invasive and label-free; however, these approaches may constrain bioreactor design and have limited detection depth. Additional product development is needed in all of these efforts for proteins to be effectively commercialized and implemented as part of a TEMP manufacturing process.

### 3.4. Substrates and metabolites

#### 3.4.1. Overview and significance

Substrates and other small molecules provide the essential nutrients to fuel cellular metabolism, and metabolites can be used as metabolic process indicators. The primary small molecules of interest are glucose, glutamine, and other amino acids as substrates, and lactate and ammonia as metabolites. Data from these liquid-phase measurements, especially when available at high frequency via an in-line or on-line device, can be used to achieve high-quality manufacturing by better controlling substrate availability at optimal levels to maximize outcomes such as proliferation rate, cell density, or extracellular matrix production. In contrast, typical off-line measurements are infrequent, leading to large fluctuations between conditions of starvation and excess, which is detrimental to cellular physiology and thus to cell growth, productivity, and consistency [147]. In a related way, it is important to monitor lactate and ammonia concentrations as indicators of cell health and to maintain these waste products at levels that are not inhibitory. Higher concentrations of these metabolites may indicate environmental stress on the metabolic health of cells and shifts in the concentration ratios of any of these small molecules may indicate contamination. Specifically for tissue applications, the available concentration of substrates is known to drive cell phenotype, where cartilaginous and ligamentous tissues thrive at lower concentrations of essential nutrients versus a highly vascularized tissue that demand high and continuous nutrient supply [148, 149]. In-line detection of essential nutrients is imperative to achieve optimal cellular performance and finely control cell differentiation.

#### 3.4.2. Sensor technologies

As is the case with protein sensors, technologies for the measurement of small molecule concentrations can be divided into two groups—spectroscopic-based and chemical-specific—depending on the number of analytes monitored by the sensor.

The spectroscopic-based methods discussed in the protein section are also used to detect substrates and metabolites [150, 151]. While the approaches are similar in that they use the same technology and integration into the reactor is the same, there are some analytical differences. In the field of metabolomics, there are additional techniques that can be used to determine metabolite profiles that cannot be used in proteomic approaches. For example, non-specific or class-recognition sensors, such as sensors employing impedance [152], chemoresistor [153], catalytic [154], and field-effect transistors [155], are used with ML to develop a ‘fingerprint’ toward diagnostic profiles [118, 156, 157]. In these examples, there were attempts to either identify diagnostic information based on the signal output including specific analyte or metabolite identification. This technology remains to be fully characterized for use beyond academic research environments.

As described for protein sensors, chemical-specific small molecule sensors employ a specific recognition element, so each analyte requires a different device [118, 156]. Many of the sensing technologies use a common platform that can be modified to detect different analytes, such as chemosensors (with chemical recognition elements) [158] and biosensors (with biological recognition elements) using either binding [159] or catalytic detection [160–163]. The most common chemical-specific sensor is for glucose monitoring; these typically use an enzyme, glucose oxidase, for the specificity of the sensor. Where small molecule sensors differ from protein-specific sensors is that chemical recognition elements (recognition elements that are not biologically derived) can be used.

The advantages of chemical-specific sensors for small-molecule monitoring include their specificity to the target analyte, high accuracy and precision, relatively simple operation, and moderate cost. Calibration of the at-line versions of these sensors is straightforward. The limitations vary with the technology and type of interface, but sterilization of the chemical and/or biological components of the sensor (for in-line use) or the bioreactor interface (for at-line use) is a general concern. These sensors are currently available in at-line formats with in-line, *in-situ* formats in development (e.g. BioPat Trace^®^ by Sartorius, BioProfiler^®^ Flex2 by NovaBiomedical, 2950D Biochemistry Analyzer by YSI, Cedex Bio Analyzer by Roche). Current at-line formats require lengths of tubing to transfer the sample from the bioreactor to the analyzer, which might not be desired in a manufacturing environment. In-line sensors can readily be integrated into a manufacturing process as long as there are available ports in the bioreactor vessel. These sensors can also be integrated into a flask through the cap or via an on-line flow cell in the medium circulation loop (e.g. Applied Biosensors InSens4^™^, OptiEnz Sensors, CITSens Biosystem, Jobst Technologies). Not only can these sensors detect specific substrates and metabolites in real-time, they are also self-contained and therefore do not require frequent maintenance and expensive reagents like many of the at-line metabolic analyzers. Sensor lifetime may be an issue, depending on the detection element and the cultivation duration. In general, the current commercial options for chemical-specific sensors achieve the criteria for accuracy, specificity, range, and precision but require improvements in lifetime, stability, calibration, and ability to be used in an in-line format.

#### 3.4.3. Outlook

Research and development are underway to address the current challenges of small-molecule sensors. For chemical-specific sensors, there are efforts to develop in-line sensing approaches that are sterilizable in the same manner as the bioreactor or to develop automated sampling for at-line analysis that is less cumbersome than current methods. Advances in biomimetic sensing [164, 165], which mimic biological catalysis and binding without biomolecules, are a promising solution if they can be developed to meet the requirements of metabolite sensors. For non-specific sensors, the primary focus is to improve the algorithms for data analysis to reduce the amount of training data required and to increase the transparency of the algorithm. The latter effort could involve explainable AI [166]. Lower-cost systems would allow wider application, including in R&D settings. As nutrient sensors, such as those for glucose and lactate, have become more available there are ongoing efforts to develop PAT systems that exchange media to control glucose and lactate around a set point.

### 3.5. Gases and volatile organic compounds

#### 3.5.1. Overview

Gases and VOCs are important physiological indicators and modulators of the functional properties of living systems. These sensors detect analytes reporting on functions ranging from cellular respiration, to chemical signaling, to metabolic indicators of systemic or organ specific health. Understanding and monitoring cellular respiration through systemic gas exchange can be achieved through the assessment of concentrations of O_2_ consumed and CO_2_ released during cellular respiration. Dissolution of CO_2_ in aqueous solution releases hydrogen ions as part of carbonic acid, lowering the pH. Bioprocess monitoring and control often uses pH detection methods (described in section 3.6) to infer and direct CO_2_ levels. O_2_ must be carefully controlled, and therefore measured in real-time, not only for the essential role in metabolism and respiration, but also because the concentration of O_2_ has been shown to drive cell behavior including differentiation and proliferation [167–169]. In bioreactors, closed-loop control systems continuously monitor dissolved oxygen (DO) levels and rely on a PID controller connected to a gas injection system to maintain target DO levels. TEMP manufacturing typically leverages DO sensors during the cell expansion and tissue/organ maturation phase. However, monitoring and control during scaffold fabrication and storage/transport with suitable form factor sensors may provide quality assurance data, especially for oxygen-sensitive cell types. Other gas sensors are more likely to be used for real-time monitoring of process specific attributes versus a continuous feedback loop for set point maintenance.

#### 3.5.2. Sensor technologies

##### 3.5.2.1. Oxygen

DO concentration is highly important in cell culture and affects many critical cellular outcomes such as viability, migration, proliferation, and differentiation [167–170]. Along with pH, DO is one of the most commonly monitored physicochemical parameters in biopharmaceutical applications and impacts scale-up and scale-down bioprocessing. Detection technologies include iodometric (e.g. colorimetry and spectrophotometry), electrochemical (current-type, conductance-type, and potentiometric type), and optical methods [12, 171]. While highly accurate, iodometric detection is difficult to execute in continuous real-time detection and will not be discussed in this section. Electrochemical and optical methods are more widely used for continuous and dynamic detection in a process.

A long-established approach for measuring DO relies on the use of the Clark-type electrochemical sensor, allowing for the direct quantification of DO levels in biological fluids. In these electrode or polarographic sensors, the chemical reduction of molecular O_2_ on a polarized platinum cathode generates a current proportional to the concentration of DO present. Recent miniaturization of Clark oxygen sensors have led to microscale electrochemical devices amenable to continuous in-line DO monitoring in tissue culture. Commercial options encompass electrode architectures with tip diameters on the order of 10 *μ*m (Unisense) and flow cell configurations capable of achieving continuous monitoring (Strathkelvin Instruments). The use of electrode sensors has decreased in recent years because electrodes consume O_2_, which is especially problematic when measuring very low levels of O_2_, and they require relatively frequent calibration.

Optical sensors have emerged as an attractive alternative and rely on either fluorescence quenching, phosphorescence quenching, NIR, or absorption [172]. The majority of these optical sensors rely on quenching of the luminescence of an indicator dye by molecular oxygen. Optical DO sensors can be inexpensive, are easy to miniaturize, are virtually non-invasive, and do not consume oxygen to take a measurement. These sensors are typically placed inside a culture vessel and require a reader for measurements. The reader might be positioned outside the vessel across from a sensor through an optically clear material or inside the vessel in the form of an optical fiber probe that includes the sensor. Optical fiber reader probes may be inserted inside a bioreactor through a port or be placed inside a flow-through cell within a perfusion flow loop (Scientific Bioprocessing, Pre-Sens, Hamilton, PyroScience GmbH).

While monitoring DO levels in the bulk cell culture medium provides critical insights into the conditions cells are experiencing, 3D tissues and organs challenged by limited oxygen diffusion with a typical diffusion limit of ~200 *μ*m. Real-time measurements of DO levels and gradients within 3D tissues and organs noninvasively are challenging but some technologies are emerging to provide much-needed insights [173–178]. A non-destructive and non-invasive 3D oxygen imaging technique using electron paramagnetic resonance is able to measure oxygen deep in tissues both *in-vivo* and *in-vitro* (e.g. JIVA-25^™^ by O2M), however, this is an at-line approach requiring the tissue or subject to be transferred into the instrumentation for testing [173]. Some approaches are being designed for implantation to continuously monitor tissue oxygenation and are being tested in animals and humans [178].

##### 3.5.2.2. Other gases

Besides the respiratory function of O_2_ and CO_2_, gaseous signaling molecules regulate cardiovascular, immune, and other functions of living systems. These signaling molecules include gasotrasmitters, such as NO, CO, H_2_S, as well as other gases, such as NH_3_, CH_4_. Electrochemical sensors for NO and H_2_S are commercially available (e.g. NO and H_2_S microsensors by Unisense), however, differentiation and selectivity in the detection of gaseous signaling molecules from each other and from common biological interferents remains an ongoing challenge [179]. With recent development, novel multiplexed electrode configurations capable of detecting multiple gaseous signaling molecules simultaneously using tailored electrode design point to future design strategies for enhanced selectivity, compared to individual sensors [180]. Challenges associated with selectivity in complex media in the presence of a multitude of interferents will need to be strategically resolved to enable continuous multiplexed monitoring of gaseous signaling molecules in live tissue. Other methods of measuring gases include IR absorption spectroscopy, Raman spectroscopy, piezoelectric absorption, and mass spectrometry. However, compared to the methods outlined above, these techniques require highly specialized instrumentation, have relatively large size, and may have challenges with spatiotemporal resolution.

Sensors positioned in the bioreactor headspace for the detection of VOCs could enable metabolic fingerprinting of gaseous and volatile biomarkers of cell phenotype, tissue maturation state, or bacterial and fungal infections. Cell specific behavior or bacterial and fungal infections produce distinct patterns of gaseous and volatile chemicals that are released into the headspace of culture [181–183]. Electronic and optoelectronic nose sensor arrays can accurately detect the identity and concentration of VOCs by combining several sensor elements to create a ‘fingerprint’ through pattern recognition [155, 184]. Recent advancements have adapted the analytical principles underlying the traditional off-line thermo-desorptiongas chromatography mass spectrometry (TD-GC-MS) analytic approach into a chip-based format called high asymmetric longitudinal field ion mobility spectrometry (HALF-IMS), which allows separation of ions based on mobility differences in high and low electric fields. Rapid data analysis combined with a learning algorithm is embedded into the device to achieve the optimum detection capabilities as portable trace chemical detectors that can be integrated into a culture system [185]. HALF-IMS technology successfully measured VOC emissions from cell bioreactor gas exhaust lines to distinguish between changes in cell cultures over time such as cell types, cell density, and biomarker dynamics over time [186]. While these technologies are still in early phases of development, they offer an exciting alternative approach to non-invasively metabolically profile cell expansion and phenotype.

#### 3.5.3. Outlook

With ample commercial options for the detection of DO, a key consideration for DO sensors is whether they need to be reusable or single-use for a process. Reusable sensors require calibration, mounting, sterilization, and manual integration, whereas single-use sensors reduce contamination risk and eliminate cleaning and validation. However, the adoption of single use DO sensors has been slow due to concerns about drift, stability after irradiation, and sensor life-time. Optical sensors are a good fit for single use systems such as high throughput microbioreactors and in single use bag bioreactors [187–189]. Remaining challenges in optical sensing in media include further performance improvements for long-term use, gamma irradiation compatibility, wireless sensing, and low cost for single use applications.

Most commercial oxygen detection sensors are intended for the detection of bulk media using either a small extracted volume or a single point within a large media bath, but not designed to detect oxygen within the tissue surface or depth. With large volumes of media, the user should be cognoscente of potential analyte gradients and placement of the sensor. The solutions being developed will be valuable tools for modeling oxygen diffusion and uptake to optimize and control oxygen delivery throughout the tissue. Tissue-integrating microsensors where the sensor is embedded throughout the tissue depth to obtain oxygen microenvironmental information are in research and development [175–177]. Sensors integrated into the tissue would have limited use to process development and product design since they would be an undesirable component in a TEMP for implantation. While some of the sensors could remain in the tissue product, they will face heavy regulatory scrutiny which may be seen as an unnecessary burden.

Beyond oxygen, while sensors for CO_2_, NO, and H_2_S are commercially available, 3D imaging, mapping, and selectivity of detection remain ongoing challenges that require dedicated effort for broad applicability to tissue engineering. While research into the metabolome is still under current investigation, studies suggest that monitoring of signature VOCs in cell culture using methods of mass spectrometry and chemical sensor arrays can enable the detection of disease states in such settings [190].

### 3.6. pH

#### 3.6.1. Overview and significance

Regulation and the stability of the proper pH balance are essential as deviations from an optimal pH may alter cell metabolism and lead to apoptotic cell death [191]. pH is a non-dimensional value measuring the degree of acidity or alkalinity of an aqueous solution. As defined by american society for testing and materials (ASTM), pH is the negative logarithm of the hydrogen ion concentration in a liquid (in moles/liter) [192]. In most mammalian species, the normal *in vivo* pH of the cells and their environment is 7.2–7.4 [193], and pH deviations from the target range or rapid changes may be lethal or impact cellular behavior [191]. In TEMP manufacturing, pH measurements may be necessary during culture media preparation, cell expansion, tissue/organ maturation and TEMP storage. Buffering systems help maintain pH within target levels to some extent [194] but closed-loop control with acid/base or carbon dioxide dispensing may be needed to maintain pH of metabolically active cell cultures. In- or on-line pH detection is necessary to maintain physiologic conditions throughout the production of cells, tissues, and organs. Beyond being used to maintain a CPP, pH can be used to indicate critical changes in the culture, such as a cell metabolism, culture contamination, or a change in cell viability [124, 194].

#### 3.6.2. Sensor technologies

At the basic research scale, cell culture media often contain Phenol Red pH visual indicator dye that is sensitive to pH changes in the range of 6.8 (yellow) to 8.4 (purple) [195]. Small-scale production typically relies on off-line measurements. However, such an approach is labor-intensive, increases contamination risk and does not necessarily deliver the required accuracy [196]. When pH data are used for closed loop control, sensor accuracy, stability, data sampling frequency, minimal drift, and resistance against fouling are some of the key parameters that ensure operational reliability. The technologies for the measurement of pH amenable to manufacturing integration fall into two groups—electrodes and optical sensors.

A pH electrode meter measures the electrical potential difference (voltage) between an indicator electrode that is sensitive to hydrogen ions and a reference electrode that carries a known electric potential immersed in the test solution [197]. The glass indicator electrode is calibrated using standard buffer solutions of known pH. Since the potential developed across the pH indicator glass electrode membrane is temperature-dependent, some pH meters are equipped with automatic temperature compensation. Electrodes are durable and may be reused for small micro-measurements (e.g. in multi-well plates) or large-scale measurements (e.g. 1001) in a bioreactor. Age, condition, calibration and cleanliness of the probe may affect pH measurements. These types of probes are widely used to measure pH off-line, but the measurements are labor-intensive, slow and may not be representative of the original system due to possible drift caused by CO_2_ off-gassing. Electrode probes are also used in-line in sterile bioreactor cultures and are mostly attached by threading through a port with a standardized PG 13.5 connection (e.g. Hamilton, Mettler Toledo, Zimmer and Peacock Ltd, PendoTECH, Endress + Hauser, Inc.). Sterilizable probes are compatible with clean-in-place and sterilize-in-place processes. Some of the performance issues include membrane fouling, electrolyte refilling, measurement drift, and the need for frequent recalibration. Most electrode pH probes are reusable; however, gamma-irradiated, single-use probes are emerging as an alternative.

Optical pH sensors are based on organic dye molecules with pH-dependent spectral properties. The loss or gain of a proton changes the electronic structure of the molecule, producing a measurable change in the way the molecule interacts with light: absorption of light at a particular wavelength, or fluorescence by one form of the molecule which may be detected spectrophotometrically [197]. Fluorescent optical sensing requires two essential elements in close proximity to each other: the fluorescent dye that is typically packaged in a hydrogel material and a reader for excitation and detection. Optical sensors are minimally- or non-invasive and non-destructive methods to perform continuous, real-time, scalable and automated pH measurements. Optical pH sensors are commercially available in a wide range of form factors: as immersible probes, sensor spots that attach directly to the interior of a culture vessel, and flow-through cells for use in perfusion flow loops (e.g. Scientific Bioprocessing, SAFE Sens, Pre-Sens, PyroScience GmbH, Ocean Insight). In probes and flow-through cells, the sensor and reader are typically packaged together using fiber optic cables or wireless readers, whereas sensor spots allow more flexibility in reader selection but still require optically clear vessel wall material for measurement. The advantage of pH optical sensors is their form factor and non-invasive nature in the cell culture processes that may achieve measurements from microliter up to m^3^ scale. Another advantage of optical pH sensors is that they may come pre-calibrated and require minimal maintenance since they are designed to be single-use and disposable. Although optical microneedles may achieve exact localization of the sensor tip inside the sample and may measure micro-volumes, the sensor tips may be fragile and easily damaged and may experience drift and stability issues in long-term culture.

#### 3.6.3. Outlook

Commercially available sensors meet many of the industry requirements and product solutions abound to achieve the vision of PAT. Technologies exist to ensure that pH fluctuations are known and accounted for from early cell expansion to TEMP bioreactor culture and even during transport, resulting in more consistent product. In biomanufacturing, the probes that take measurements from one location in a bioreactor may miss inadequate fluid mixing and yield in pH variability within a culture vessel. The emergence of pH sensor spots coupled with fiber optic reading devices provides more placement flexibility and allows for pH mapping within a culture vessel. Optical sensors can measure pericellular pH due to their form factor and can be attached directly to a cell culture surface where the cells are growing [198]. In bioreactors, multiple optical sensors can be used to map the pH distribution without the need for multiple bioreactor ports. While optical sensors offer great promise for cell, tissue, or organ manufacturing, their broad use is limited by long-term stability challenges. While optical sensors have significantly improved drift over electrochemical probes, manufacturing processes may run for several months, which even at a low drift of 0.005 pH per day that some optical sensor products report can be problematic.

Recently, intracellular pH sensors have also emerged. Intracellular pH (pH_i_) is tightly controlled but there is variation in pH_i_ levels between cell types and tissues as well as between different organelles and the cytoplasm (<5.5 in lysosomes, ~7 in the endoplasmic reticulum and ~6 in the trans-Golgi network) [199]. While it was previously thought that intracellular pH remains mostly constant, new studies have emerged showing intracellular pH fluctuations, and its role in cancer and stem cell proliferation and differentiation [200, 201]. Fluorescence [202–204] and luminescence [205–207] are two of the main methods used to measure pH_i_, and commercially available pH_i_ indicators (e.g. ThermoFisher Scientific, Sigma-Aldrich, abcam) track the internalization of fluorophores in the cytosol or in particular organelles. To date, pHi is mostly measured in the research and development stage.

## 4. Discussion and conclusion

Depending on the quality attribute intended to be measured, there is a breadth of sensor options to select from that will inform the user’s knowledge about that attribute. Cell culture environment sensors for pH, temperature, and DO are widely available and for the most part suit the requirements for tissue manufacturing. These sensors are relatively common as part of a PAT to monitor and control around a set point. Beyond just environmental control, these tools could be further leveraged as indicators of cell and tissue health since the consumption and availability of ions and gases are critical components of cellular respiration. Increasingly, analyte-specific sensors for metabolites and proteins are becoming available, particularly for glucose and lactate, but face challenges in maintaining performance metrics after sterilization and longer duration. In-line, non-destructive, and real-time detection becomes more challenging as the measurement target gets more complex. While tools are available, the cost-benefit balance shifts because many of these tools either suffer from low-throughput or are challenging to integrate into a manufacturing workflow.

This review presents sensors that are currently or soon to be available. These sensors can be correlated as indicators of CQAs such as cell health, cell identity, and tissue maturation and leveraged to establish in-line tissue quality control. The complexity of the cell and tissue products being manufactured and the breadth of measurement outputs means that one sensor will not be sufficient. However, the use of a QbD approach enables the developer to utilize well-designed experiments in tandem with powerful discovery measurement tools to correlate CQAs with in-line sensors for real-time quality control of the tissue product. For example, glucose and lactate sensors can be used to indicate cell health, including metabolism and proliferation. Or detection of a secreted protein can indicate cell identity or tissue matrix content. By establishing a limited set of CQAs to detect, the benefits of in-process quality control should out way the costs of either developing a model for optical sensors or a specialized chemical sensor.

While the existing sensors can be leveraged for improved tissue quality control, in many respects these technologies fall short of the needs of the industry. Cost and lack of knowledge continue to be major barriers to the adoption of the sensor in manufacturing. Non-specific sensors, such as optical approaches, are incredibly powerful measurement tools. These approaches need to be further developed to bring down the cost of interfacing these tools with multiple bioreactors in an automated workflow and the in-house expertise required for implementation. It is worth noting that costs can be deceptive for non-specific sensors such as Raman and NIR. The upfront costs may be high and end-users should view these sensors as a long-term investment since these technologies can be integrated as part of a PAT and can be adapted to multiple targets [134]. Conversely, target specific sensors, such as protein biosensors, have the advantage that they are low-cost and specific, but they are typically single-use and at-line. Current commercial products are aimed at qualifying measurable attributes secreted into the culture media or in the bioreactor headspace, requiring assumptions to be made about what is happening inside the developing tissue. This is true for many of the sensors presented here. Commercially available products for in-line non-destructive assessment of oxygenation, nutrient delivery, metabolic activity, and protein content beneath tissue surface are a much needed manufacturing tool. Furthermore, tissue processes are much longer than adjacent application in biopharma. Sensors capable of long-term duration detection (>7 d) will be necessary to achieve the in-line vision as part of a PAT. Developers should always bear in mind the opportunity cost, in-line sensors as part of a feedback driven process are required to achieve the vision of a flexible manufacturing process, realize a reduction in production costs, achieve product consistency and quality, and reduce the risk of failure.

Translation of novel sensors requires collaboration between sensors and measurement developers, cell and tissue end-users, and automation and equipment experts. Many countries have set up nonprofit consortia intended to support technology development projects and foster cross-disciplinary relationships (i.e. U.S.- BioFabUSA, NIIMBL, Cell-Met, CMaT, U.K.-Catapult, Canada—CCRM). These institutes serve as centralized hubs to connect manufacturing resources and create a collaborative environment for new technology advancement. Furthermore, developers need to consider the potential regulatory implications of a novel approach to the characterization of regulated product. Method validation strategies will need to be in place to meet late-stage regulatory needs. In the US, the FDA has established programs such as CDRH’s Medical Device Development Tools Program and CDER’s Emerging Technology Program. More recently, CBER established the CBER Advanced Technologies Team (CATT) Program. Through the CATT program, prospective innovators and developers of advanced manufacturing and testing technologies for cell therapies and TEMPs can interact and discuss with CBER staff the implementation of these technologies. The development and adoption of consensus standards is going to be critical for the integration of sensors as part of a PAT. The Standards Coordinating Body in the United States helps to coordinate regenerative medicine standards across standards development organizations and connects experts to accelerate development timelines. Consortia provide venues for pre-competitive public-private partnerships in place as a means to engage with government organizations on manufacturing relevant topics such as standards, analytical methods, and regulations.

Cell, tissue, and organ technologies are changing the landscape of medical treatment options. The current manufacturing approach needs in-line, non-destructive, and non-invasive tools that can integrate into the manufacturing process to monitor product quality in real-time and enable process efficiency and decision making. The implementation of these sensors will be the linchpin for scalable, modular, automated, and closed manufacturing.

## Figures and Tables

**Table 1.** Example quality attributes associated with a measurable characteristic.

Measureable attribute	In-process quality attribute examples
Tissue structure and function	Elastic modulus, viscosity, contractility, conductivity, matrix organization
Cellular properties	Viability, morphology, motility, confluence, cell number, cell health, cell identity
Protein expression (proteomics)	Matrix production, cell differentiation and phenotype, cell health
Cell metabolism (metabolomics)	Nutrient and waste analysis, cell signaling, cellular bi-products, cell health
Lipid production (lipidomics)	Cell differentiation and phenotype, cell health
Gene expression (genomics)	Cell differentiation and phenotype, cell health, genomic stability
Gas and VOC	Respiratory activity, oxygen and carbon dioxide transfer rates, sterility
Culture environment (pH, osmolality, temp, media composition)	Cell health, sterility

## Data Availability

All data that support the findings of this study are included within the article (and any supplementary files).

## Terminology

**Table:**

Term	Definition
*General Terms*	
Sensor	A device that measures a physical quantity and converts it into a signal which can be read by an observer or by an instrument. A sensor is a device, which responds to an input quantity by generating a functionally related output usually in the form of an electrical or optical signal [1].
Measurement	The process of data collection, analysis, and reporting [2].
*Process Development Terms*	
Critical quality attribute (CQA)	A physical, chemical, biological or microbiological property or characteristic that should be within an appropriate limit, range, or distribution to ensure the desired product quality [3, 4].
Critical process parameter (CPP)	A process parameter whose variability has an impact on a CQA and therefore should be monitored or controlled to ensure the process produces the desired quality [3, 4].
Quality by design (QbD)	A systematic approach to development that begins with predefined objectives and emphasizes product and process understanding and process control, based on sound science and quality risk management [3, 4].
Process analytical technology (PAT)	A system for designing, analyzing, and controlling manufacturing through timely measurements (that is, during processing) of critical quality and performance attributes of raw and in-process materials and processes with the goal of ensuring final product quality [3, 4].
Quality target product profile (QTPP)	A prospective summary of the quality characteristics of a drug product that ideally will be achieved to ensure the desired quality, taking into account safety and efficacy of the drug product [3, 4].
*Measurement Location Terms*	
In-line	Measurement in which the sample is not removed from the process stream [5].
On-line	Measurement in which the sample is diverted from the manufacturing process to an analytical instrument and may be returned to the process stream [5].
At-line	Measurement in which the sample is removed, isolated from, and rapidly analyzed in close proximity to the process stream [5].
Off-line	Measurement in which the sample is removed, isolated from, and analyzed in an area remote from the manufacturing process [5].
*Sensor Attributes Terms*	
Destructiveness	The extent to which a method used to detect and evaluate quality attributes in a material or system impacts the overall process. A non-destructive measurement would have minimal interaction with the target and would not disturb or invade the biological operation of the system.
Invasiveness	The extent to which a method used to detect and evaluate quality attributes in a system poses a contamination exposure risk during use. A non-invasive measurement would not require breaching the sterile boundary of the culture system.
Label-free	The molecule of interest is not extrinsically marked or tagged in order to measure it.
Real-time	A measurement occurring on demand or continuously with response time much less than the dynamics of the system being measured.
Single-use	Sensor is intended for one-time use, and cannot rapidly and reproducibly be regenerated. A disposable component containing a one-time use biorecognition element inserted into a multi-use analytical instrument is considered single-use.
Multiple-use	Sensor is used multiple times or contains a regenerative component that can be used for the duration of the process. A multiple-use sensor is used for the duration of a single batch and disposed of after a defined number of uses or duration of use.
Reusable	A sensor that can be used for an ‘unlimited’ number of uses or across multiple batches.
Automated	Requires no human intervention to conduct measurement.
